# Measles recognition during measles outbreak at a paediatric university hospital, Austria, January to February 2017

**DOI:** 10.2807/1560-7917.ES.2020.25.3.1900260

**Published:** 2020-01-23

**Authors:** Benno Kohlmaier, Nina A Schweintzger, Werner Zenz

**Affiliations:** 1 Department of General Paediatrics, Medical University of Graz, Graz, Austria

**Keywords:** measles recognition, nosocomial measles transmission, measles outbreak, atpyical measles, measles outbreak costs analysis

## Abstract

Recognition of measles is crucial to prevent transmissions in the hospital settings. Little is known about the level of recognition of measles and possible causes of not recognising the disease by physicians in the post-vaccine era. We report on a measles outbreak in a paediatric hospital in Austria in January to February 2017 with strikingly high numbers of not recognised cases. The extent and course of the outbreak were assessed via retrospective case finding. Thirteen confirmed measles cases were identified, two with atypical clinical picture. Of eight cases with no known epidemiological link, only one was diagnosed immediately; four were recognised with delay and three only retrospectively. Eleven typical measles cases had four ‘unrecognised visits’ to the outpatient clinic and 28 on the ward. Two atypical cases had two ‘unrecognised visits’ to the outpatient clinic and 19 on the ward.

Thirteen clinicians did not recognise typical measles (atypical cases not included). Twelve of 23 physicians involved had never encountered a patient with measles before. The direct and indirect costs related to the outbreak were calculated to be over EUR 80,000. Our findings suggest the need to establish regular training programmes about measles, including diagnostic pitfalls in paediatric hospitals.

## Background

Despite elimination efforts, a considerable increase of measles cases in the European Union/European Economic Area (EU/EEA) countries was observed between January 2016 and March 2019 compared with previous years, with 44,074 measles cases being reported. Countries most affected were Romania, Italy, France, Greece, Germany and the United Kingdom [1].

Main risks for outbreaks include low vaccination coverage, importation of measles and nosocomial spread [2,3]. In a number of outbreaks, hospitals were amplifiers and healthcare workers (HCW) were infected [4,5]. Major reasons for measles transmission in hospitals are the high contagiousness of the measles virus, the capacity of the virus to persist in aerosol suspensions, unvaccinated healthcare personal, the nonspecific initial presentation of the patients, crowding of patients in outpatient clinics, inability to isolate febrile children from afebrile children in waiting rooms and the lack of awareness of physicians [2,6-9].

Typical measles symptoms include a prodromal stage with fever and upper respiratory symptoms, including coryza, conjunctivitis and a dry cough. After 2–4 days, a maculopapular rash starting from the face spreading down the body appears. The rash gradually recedes, fading first from the face and last from the thighs and feet. However, some patients might present with atypical symptoms, e.g. the rash might not start on the face or not be maculopapular (e.g. be purpuric instead). Patients with atypical measles symptoms or not presenting with full symptoms of the disease contribute to misdiagnoses during outbreaks [10,11].

Since 2015, Austria recommends the first dose of measles-mumps-rubella (MMR) vaccine at 9 months of age; however, MMR vaccination can be started at 6 months of age during a measles outbreak. First dose MMR vaccination coverage in children 2–5 years is 95%, but second dose coverage is only 84% [12].

## Outbreak detection

In January 2017, we noticed a measles outbreak at the Department of Paediatrics and Adolescent Medicine with six cases occurring within 2 weeks, all without a known source of infection. Here we give a detailed outbreak description, including possible reasons for clinicians not recognising measles.

## Methods

We performed a retrospective analysis of all patients visiting the Department of Paediatrics and Adolescent Medicine, Medical University of Graz from January to March 2017 to describe the measles outbreak in early 2017.

We adhered to World Health Organization (WHO) definition by declaring a measles outbreak as two or more laboratory-confirmed cases that can be epidemiologically or virologically linked [13]. The outbreak time frame was defined from time of symptom onset of the first case until 21 days after the last case was diagnosed.

### Case definition and genotyping

We used the European Centre for Disease Prevention and Control’s (ECDC) measles case definition [14]. Measles infection was verified using real-time PCR (FTD Measles, Fast Track Diagnostics, Sliema, Malta) on throat swabs or ELISA (Enzygnost Anti-Measles Virus IgM and IgG, Siemens Healthcare Diagnostics, Marburg, Germany) on sera. Throat swabs were sent to the National Reference Centre for Measles, Department of Virology, the Medical University of Vienna for confirmation and strain analysis. Genotyping was performed according to the measles and rubella WHO reference laboratory recommendations [15] using the Measles Nucleotide Surveillance (MeaNs) database tool for sequence analysis of a 450 nt amplicon coding for the nucleoprotein (N-450).

The outbreak description included all patients with confirmed measles that were seen in our paediatric university hospital. Patients were numbered according to symptom onset.

Information on the number of reported measles cases in the district of Styria, Austria from 2009 to 2017 was provided by the Landessanitätsdirektion Graz, Austria.

### Measles recognition

The analysis of measles recognition included all patients with confirmed measles and maculopapular rash, and excluded all patients with a known epidemiological link or referral with suspicion by a general practitioner or extramural paediatrician.

Diagnoses were categorised as ‘immediately’ if a measles patient was recognised at first presentation in exanthema stage or earlier, ‘delayed’ if a patient had at least one unrecognised visit in exanthema stage, and ‘in retrospective’ if a patient was diagnosed after acute measles during the retrospective outbreak investigation.

Retrospective data collection and analysis of medical records of measles cases was undertaken using the electronic documentation system openMEDOCS, containing information about all patients presenting at our hospital. Furthermore, we queried the database for measles notifications transmitted to the Austrian measles registration system from 27 January to 1 March 2017. Data collection and analysis were extended to 21 days after the last measles case was confirmed at our hospital in order to cover the maximum known incubation period [16]. Information on arrival and departure times of outpatient visits as well as admission times on the ward were collected in addition to immunisation status and demographic, epidemiologic and clinical information. Exposure was deemed to have occurred if a person was present with the source case, i.e. the primary case (P1), during an outpatient visit. We gathered information about the transmission routes by interviewing the patients’ families.

For those patients who presented in exanthema stage and without a known epidemiological link or referral, the number of out- and inpatient visits where measles was not recognised together with the number of all involved clinicians was analysed.

We investigated the possible reasons for not recognising measles via a detailed review of all cases by two independent clinicians. The review procedure included a summary of all available medical documents and personal interviews with the treating clinician.

### Measles experience of involved clinicians

We used an interview-delivered survey to collect data on the individual measles experience of clinicians involved in the management of the measles patients. In the interview, we asked about the total number of measles patients they had seen before and the length of time they had been practising medicine.

To investigate the economic impact of the not recognising measles of this outbreak, we analysed the direct and indirect costs related to it. The data collected included the costs of outpatient visits [17], costs of hospital stay on the general ward (as calculated by our hospital finance department), costs for antibody and PCR testing, costs associated with the estimated working hours for outbreak management and indirect costs from the productivity loss of parents, patients or both. The latter were calculated using a report about Austrian absences [18].

We investigated a measles outbreak in 2019 to compare our findings with this later outbreak. We analysed the visits of all cases in exanthema stage without a known epidemiological link or a referral. The 2019 outbreak was also from January to February and analysed in the same way as the 2017 outbreak. The total number of antibody and PCR tests was included in the analyses.

### Statistical analysis

All data were anonymised and entered into a Microsoft Access database. Data were analysed using Excel and descriptive statistics.

### Ethical statement

This study was approved by the local ethics committee of the Medical University of Graz (EK 30–062 ex 17/18). We have conducted this study consistent with the Declaration of Helsinki.

## Results

Thirteen measles cases presented to our Department of Paediatric and Adolescent Medicine between 13 January 2017 and 8 February 2017. All cases were laboratory confirmed. There were nine female and four male cases, with a median age of 12 months (range: 2 months–30 years). Six cases were infants under 1 year of age. Six cases became infected after visiting the outpatient clinic and two while hospitalised. One was a paediatrician in training. Eleven of 13 cases were unvaccinated, including three infants not eligible for routine vaccination. One case had received only one dose of MMR vaccine 9 years earlier. The paediatrician in training was fully vaccinated but received immunosuppressive treatment as a child.

### Outbreak description

On 27 January 2017, the first measles case was diagnosed (index case, patient (P)5). Three days later, an additional three cases (P3, P4 and P6) were diagnosed. Five days after the first case, another two cases (P7 and P10) were diagnosed. None of the cases had a known source of infection.

The outbreak investigation was initiated after the first case was diagnosed, and discovered that three cases were treated in the outpatient clinic on 13 January 2017 for minor illnesses. By reviewing the medical records of all patients who were present on that day, we identified a teenager as the source case (P1). Measles was not recognised and P1 came into contact with 34 patients, infecting nine children.

**Figure 1:**
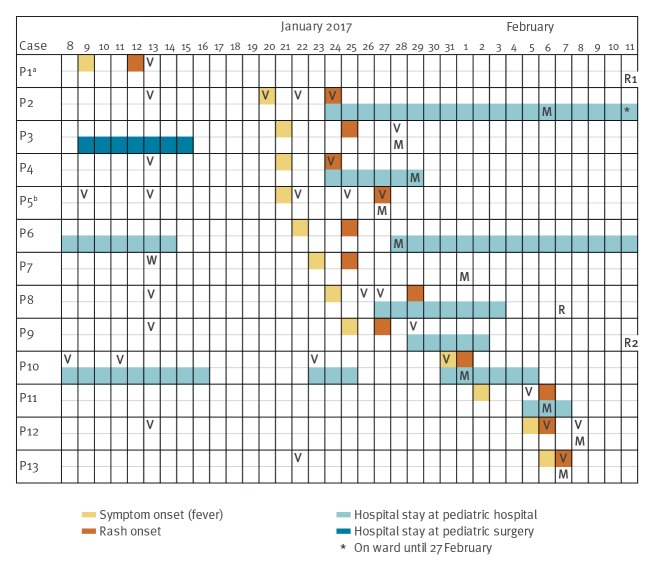
Timeline of symptoms onset, outpatient visits, hospital stays and measles testing of all measles patients, Styria, Austria, January to February 2017

**Figure 2:**
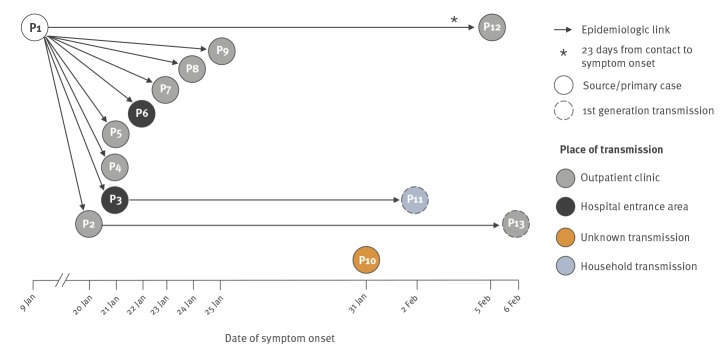
Chain of measles transmission, Styria, Austria, January to February 2017 (n = 13)

Another six outbreak cases were identified (P2, P8, P9, P11, P12 and P13), two of which were diagnosed retrospectively (P8 and P9) (Figure 1 and 2).

M: testing for measles; R: retrospective testing for measles; R1: retrospective testing on 23 February; R2: retrospective testing on 30 March; V: visit in outpatient clinic; W: working in outpatient clinic.

^a^ Source/primary case.

^b^ Index case.

P: patient.

### Patient descriptions

P1 was a teenager who presented with fever, coughing and an itchy maculopapular rash that had appeared after taking a bath with an additive. P1 was diagnosed as febrile illness and allergic reaction to the bath additive. After being clinically identified as a possible source case, P1 tested positive for measles-specific IgM and IgG antibodies by ELISA. From interviews we learned that P1 was in the outpatient clinic for 4 hours and waited to be picked up for an additional 2 hours in the entrance area of the hospital. During the incubation period, P1 had met friends who recently had stayed in Romania, where a measles outbreak was reported [19].

P2 was a 12-month-old infant. Seven days after the presentation of the source case (APSC), P2 presented twice to the outpatient clinic with fever and also tested positive for influenza A virus through rapid antigen testing. Nine days APSC, P2 came to the outpatient clinic with persistent fever and oral mucositis, and 11 days APSC, the case presented at the outpatient clinic with fever, conjunctivitis, a maculopapular exanthema, diaper mucositis, hypoxaemia and oral aphthous ulcers. The case was hospitalised and diagnosed with influenza A infection and erythema exsudativum multiforme. Twenty-four days APSC, the treating physicians took a serum sample to measure herpes simplex virus (HSV) antibodies, describing a ‘fever and rash’ on the delivery note. The hygiene institute was aware of the measles outbreak and also tested for measles IgM. HSV-1 and measles IgM were both positive. This result was confirmed by a positive measles PCR in saliva. The case was classified as HSV-1 associated erythema exsudativum multiforme and measles infection. Through review of medical records, it was determined that P2 was in contact with the source case in the outpatient clinic.

P3 was a teenager, an inpatient at the paediatric surgery department and discharged from the hospital 2 days APSC. Thirteen days after release from hospital, the case was referred to the outpatient clinic by their practitioner with suspected measles because of fever and exanthema. Measles was verified by measles-specific IgM and IgG antibodies and PCR. The patient interview revealed that P3 was in contact with the source case at the entrance area.

P4 was a 9-month-old child. Eleven days APSC, P4 visited the outpatient clinic with fever, cough, full body rash and conjunctivitis. The case was diagnosed with ‘fever without source’ and admitted to the ward where a urinary tract infection with pyuria was detected. Sixteen days APSC, clinicians suspected measles. The diagnosis was verified by measles-specific IgM and IgG antibodies and PCR. Via review of medical records, it was determined that the case had contact with the source case in the outpatient clinic.

P5 was an 11-month-old infant who was seen four times in our outpatient clinic. Nine days APSC, the case presented with fever and obstructive bronchitis, 12 days APSC, P5 presented with mucositis and tonsillitis, and 14 days APSC, P5 presented with fever and a full body rash. The case was initially diagnosed with drug eruption because of cefaclor therapy. P5 returned to the clinic the same day and was diagnosed with measles, which was confirmed by PCR. Contact with the source case in the outpatient clinic was established.

P6 was a cystic fibrosis patient in their early 20s who was referred by their general practitioner with suspicion of measles on 28 January. P6 had fever and a maculopapular rash, and was hospitalised for 15 days. Measles was confirmed by PCR. The case was an inpatient when the source case presented to the hospital and met P5 in the entrance area.

P7 was a resident in their 30s. Ten days APSC, P7 had fever and went on sick leave. Twelve days APSC, P7 had a mild generalised maculopapular rash for 3 days. Nineteen days ASPC, the case suspected themself of having measles, which was confirmed by positive IgM antibodies. P7 had been vaccinated twice against measles but had undergone immunosuppressive therapy because of a malignancy during childhood. P7 met the source case when working in the outpatient clinic.

P8 was a 7-month-old infant. Thirteen days APSC, the case visited the outpatient clinic with fever, coughing and diarrhoea, and was diagnosed with a respiratory tract infection. The next day, P8 was admitted to the ward because of dehydration. The stool PCR was positive for norovirus and a throat swab PCR was positive for respiratory syncytial virus (RSV). Seventeen days APSC, the case was described as having a full body rash in the nursing report. Twenty-one days APSC, P8 was discharged with the diagnosis of RSV-positive bronchopneumonia. The rash, or any diagnosis referring to it, were not mentioned in the discharge letter. The outbreak investigation identified the patient as a possible measles case, which was confirmed by PCR from a preserved throat swab. The case was in contact with the source case in the outpatient department.

P9 was a 6-month-old infant. Sixteen days APSC, the case presented with subfebrile temperature over the previous 2 days, rhinitis, maculopapular rash, insufficient fluid intake and bloody diarrhoea. P9 was admitted to the ward and later discharged with a diagnosis of gastroenteritis. The rash was mentioned in the discharge letter but there was no diagnosis referring to the exanthema. Outbreak investigation identified P9 as a possible measles case, which was confirmed by retrospective blood testing for measles IgM. P9 encountered the source case in the outpatient clinic.

P10 was a 2-month-old infant. Eighteen days APSC, the infant’s mother was diagnosed with measles in a different hospital (and is therefore not part of this analysis). The infant was admitted to our hospital for post-exposure prophylaxis with immune globulins. On the second day of P10’s stay, the case developed a full-body rash and tested positive for measles by PCR. Outbreak investigation revealed that P10 was an inpatient with the mother the day when the source case presented. Upon interviewing, the mother reported that they had not left the ward that day. Our investigation could not identify the source of infection.

P11 was the teenage sibling of P3. Fifteen days after the onset of the sibling’s symptoms, P11 presented in the outpatient clinic with fever. Measles was detected and confirmed by PCR and antibody testing 1 day after admission.

P12 was a 10-month-old infant. Twenty-three days APSC, the case developed a fever. The next day, the case presented in the outpatient clinic with a full-body rash and was diagnosed with viral exanthema. Two days later, P12 tested positive for measles by PCR. P12 met the source case in the outpatient clinic. The outbreak investigation could not determine any other contact to a measles case.

P13 was a 10-year-old child who presented 25 days APSC with fever and full body rash, and tested positive for measles by PCR. The child was in contact with P2 during their outpatient visit 9 days APSC.

All measles cases recovered uneventfully.

### Genotype

Viral strain analysis supported the reproduced chain of transmission. All genotyped patients (P2, P3, P5 and P8) showed the same B3–4299 genotype. These sequences have been deposited in the WHO MeaNs database and assigned a MeaNs-ID: 103299, 103767, 103768 and 106868.

### Recognition of measles patients in exanthema stage

Of 13 cases, one diagnosed themself (P7), two were referred with a suspicion of measles (P3 and P6) and two had a known epidemiological link (P10 and P11). Of the other eight, only one was diagnosed immediately (P13); four patients were diagnosed after delay (P2, P4, P5 and P12) and three were diagnosed retrospectively (P1, P8 and P9) (Table 1).

**Table 1:** Characteristics and diagnostic category of measles cases with possible reasons for delayed or not recognising measles, Styria, Austria, January to February 2017

Case	Age	Number of outpatient visits in prodromal stage	Number of outpatient visits in exanthema stage	Admission to hospital	Correct diagnosis	Laboratory confirmed	Diagnostic category^a^	Possible reason for delay or not recognising measles
P1	Teenager	0	1	No	Retrospective	IgM,IgG	Retrospective	Atypical measles
P2	Infant	3	1	Yes	On ward after 13 days	PCR, IgM, IgG	Delayed	Atypical measles
P3	Teenager	0	1	No	By referring paediatrician	PCR, IgM, IgG	Referred from paediatrician	NA
P4	Infant	0	1	Yes	On ward after 6 days	PCR, IgM, IgG	Delayed	Coinfection (UTI)
P5	Infant	2	2	No	At fourth outpatient visit	PCR	Delayed	Suspected drug eruption
P6	Early 30s	0	0	Yes	By referring GP	PCR, IgM, IgG	Referred from GP	NA
P7 (HCW)	Early 20s	NA	NA	NA	Themself	IgM, IgG	NA^b^	NA
P8	Infant	2	0	Yes	Retrospective	PCR	Retrospective	Coinfection (RSV and norovirus)
P9	Infant	0	1	Yes	Retrospective	IgM	Retrospective	Bloody diarrhoea
P10	Infant	1	0	Yes	At first outpatient visit	PCR	Known epidemiological link	NA
P11	Teenager	1	0	Yes	At first outpatient visit	PCR	Known epidemiological link	NA
P12	Infant	0	2	No	At second outpatient visit	PCR	Delayed	Incubation period > 21 days
P13	10 years	0	1	No	At first outpatient visit	PCR	Immediately	NA

GP: general practitioner; HCW: healthcare worker; NA: not applicable; PCR: real-time PCR; RSV: respiratory syncytial virus; UTI: urinary tract infection; y: years.

^a^ Excluding the specialist-in-training (P7).

^b^ P7 was part of the outbreak but was not assigned a diagnostic category because this person, a clinician, self-diagnosed.

The analysis of ‘unrecognised visits’ of measles cases revealed six visits to the outpatient clinic and 47 on the ward. However, these visits includes two patients with atypical presentations (P1 and P2) who had two visits to the outpatient clinic and 19 visits on the ward. The 23 clinicians involved in the treatment of the measles cases were either paediatricians in training or paediatric specialists. In total, 18 clinicians did not diagnose measles, including eight paediatricians in training and 10 paediatric specialists. Excluding atypical cases (P1 and P2), 13 clinicians did not diagnose measles, including five paediatricians in training and eight paediatric specialists.

### Possible reasons for the delayed diagnosis or not recognising measles

The retrospective outbreak investigation and interviews with all involved clinicians revealed a high number of not diagnosed patients. For P1 and P2, atypical clinical presentations were identified as the most likely reason for not recognising the disease. A specialist did not diagnose measles in P1 because of the uncharacteristic itchy rash and the bathing with eucalyptus additive.

P2 had erythema exsudativum multiforme and no typical rash. Pictures taken from the rash were reviewed and classified as atypical. According to literature research, erythema exsudativum multiforme or Steven Johnson Syndrome can appear in measles patients, but their appearance seems to be rare [20,21].

In P4 and P8, co-infections were identified as a possible reason for not recognising measles. P4 had a urinary tract infection, and P8 was positive for RSV and norovirus which would explain coryza and gastrointestinal symptoms.

The measles rash of P5 was interpreted as drug eruption from cefaclor, while P9 was diagnosed as having gastrointestinal infection because of bloody diarrhoea. Measles was not suspected in P12 because of the incubation period, which was beyond 21 days.

### Clinician experience with measles

All 23 clinicians, 11 paediatric specialists and 12 paediatricians in training, who were involved in the management of the measles patients were asked whether they had seen a measles case before. Nine of 11 paediatric specialists, but only 2 of 12 paediatricians in training had seen measles before. The median working years of paediatric specialists was 15 years (range: 6–31) while the median number of working years of paediatricians in training was 2 years (range: 1–5). The median number of measles patients seen by paediatric specialists was two (range: 1–30). One paediatrician in training had seen one patient and the other had seen five.

### Number of measles cases in Styria from 2009–2017

According to the records of the province of Styria public health authorities, there have been a total of 150 measles cases from 2009 to 2017, with a median annual incidence rate of 1.4 cases per 100,000 population (Table 2).

**Table 2:** Measles cases reported by provincial public health authorities, Styria, Austria, 2009–2017

Year	Total number of cases	Number of cases 0–17 years of age
2009	32	27
2010	2	2
2011	18	9
2012	14	9
2013	8	4
2014	8	3
2015	31	22
2016	4	1
2017	33	15

### Economic impact of the measles outbreak

The evaluation of costs directly and indirectly related to the outbreak revealed a total amount of EUR 84,463.72 (Table 3). Inpatient stays and loss of productivity totalled EUR 74,196.26. Twenty-one outpatient visits cost a total of EUR 2,983.68 while measles testing cost EUR 1,982.18, only accounting for 2% of the total costs. Forty-six patients were tested by PCR and 16 for measles IgM antibodies via ELISA.

**Table 3:** Costs of the measles outbreak, Styria, Austria, January to February 2017 (n = 13 cases)

Service	Costs (EUR)/unit	Amount	Total (EUR)
Outpatient visits	142.08/visit	21 visits	2,983.68
Inpatient stays	808.43/stay	82 stays	66,291.26
Serological testing	8.34/test	16 tests	133.44
PCR testing	40.19/test	46 tests	1,848.74
Outbreak management	66.27/hour	80 hours	5,301.60
Productivity losses of parents or patients	127.50/day	62 days	7,905.00
Total	NA	NA	84,463.72

NA: not applicable.

### Comparison with the 2019 measles outbreak

In January 2019, a measles outbreak with 18 cases was seen at our hospital. Seven cases presented without a known measles contact and without referral from a GP or external paediatrician. All cases were recognised at first presentation. Five clinicians were involved in the diagnosis of cases, including four paediatric specialists and one paediatrician in training. All but one paediatric specialist were also involved in the outbreak in 2017. During the outbreak in 2019, we tested 129 patients by PCR and 33 for measles IgM antibodies.

## Outbreak control measures

After the 2017 outbreak, there was an intensified training course about measles with three sessions that aimed to reach many hospital employees. Three educational sessions were held. The first, held as part of our weekly education sessions, was about measles in general with a focus on providing clinical information, e.g. measles symptoms, incubation period and presentation of cases. A second session was held during our weekly presentation of interesting clinical cases, which is also a lecture for students. The third session was held as part of our yearly meeting for new diagnostic and treatment algorithms. All sessions were held within 1 year of the outbreak, and all included general measles information and detailed clinical descriptions of all cases seen at our department. We also monitored the attendance of clinicians. Of 114, 71 (62%) clinicians employed in our hospital participated in at least one session.

## Discussion

This article reports on the recognition of measles cases in a paediatric department at a central European university hospital in the ‘post-vaccination era’. It shows that only one of eight cases without a known epidemiological link or without referral from GP or external paediatrician was diagnosed immediately. We retrospectively identified and diagnosed the source case during the outbreak investigation and were able to establish a transmission chain. The isolated strain was prevalent in the latest Romanian measles outbreak that began in 2016, with 19,443 reported cases, 11,217 confirmed cases and 63 deaths from January 2016 to October 2019 [22]. Strain analysis of measles cases in neighbouring provinces of Austria showed different genotypes (personal communication, Heidemarie Holzmann, November 2017), substantiating our established transmission chain [23].

Incubation periods ranged from 7 to 23 days in P12, which, while unusual, but has been reported [24].

One healthcare worker who was fully vaccinated became infected. Although they were working while in the prodromal stage for 1 day, no onward transmission was observed. Previous reports suggest low transmission rates in breakthrough infections [25]. No other hospital employee became infected which might be the consequence of a rigorous vaccination status control of hospital employees that was established after a measles outbreak in 2015. Positive vaccination status verified by record of two doses of MMR or a protective measles titre, is a mandatory requirement for new applicants at our hospital. Mandatory vaccination in health providers is a key measure to reduce the risk of nosocomial transmissions from healthcare workers [26].

Besides a lack of experience and awareness, we identified further possible causes for not recognising measles: atypical presentations, co-infections and suspected allergic reaction to medications. Two patients had atypical presentations although they were not vaccinated and were not immunosuppressed. Literature quantifying the number of patients with atypical presentations is scarce and the prevalence of such might be subject to vaccination rates. P1 presented with an itchy rash which is uncommon. A literature review revealed only one article describing an itching character of the measles exanthema [27].

P2 presented with erythema exsudativum multiforme. After positive testing for measles IgM measles were discussed, but because of the clinical picture of erythema exsudativum mulitfomre the test was considered to be false positive. However, the additional positive measles PCR substantiated the positive serology. This patient was seen by four paediatricians in training and six paediatric specialists, including a paediatric infectious disease specialist on 20 visits before diagnosing measles.

Co-infections are common in measles infections [28,29] which can mislead a clinician’s assessment of the symptoms. One case presented with a urinary tract infection and another one with bronchopneumonia caused by RSV and norovirus gastroenteritis; one measles case diagnosed with tonsillitis was treated with cefaclor and the maculopapular rash was interpreted as antibiotic-induced exanthema. A correct differential diagnosis cannot be based solely on a clinical investigation, and in cases of doubt, measles should always be considered [30,31].

Clinicians should test for measles in patients with atypical symptoms, especially during outbreaks to avoid further spread and unnecessary inpatient stays of undiagnosed cases. Moreover, our findings support a thorough testing of patients with symptoms suggestive for measles since costs for testing were relatively small compared with costs related of not recognising measles and/or diagnostic delay.

Not recognising measles cases might be because of a prolonged period free from measles. The disease has become rare in Austria, as suggested by the number of measles cases in the province of Styria (Table 2). A tendency towards a more accurate diagnosis over time was observed. The last outbreak case, P13, was the only case diagnosed without delay and P12’s diagnostic delay was shorter compared with the average diagnostic delay.

The education sessions held after the outbreak were effective and a reminder that measles should be considered in patients with fever and maculopapular rash. During the measles outbreak in 2019, there were no cases with delayed diagnosis in our hospital. The analysis of PCR and antibody testing showed a higher rate of testing, reflecting increased disease awareness.

There are several limitations to this study. The improved recognition of measles may be a combination of pre-existing clinician diagnostic skills as well as an increased awareness for measles during the course of the outbreak. Analysis of recognition of only sporadic cases would provide more accurate evidence for increased awareness. Moreover, consecutive visits of a patient are difficult to interpret as independent events, as previous visits are apparent to the treating clinicians. The cost analysis is subject to estimation and did not consider workload associated with additional vaccinations, consultations or other healthcare providers.

### Conclusion

This outbreak shows a need for repeated awareness-raising of measles for clinicians in paediatric hospitals to ensure adequate diagnosis and awareness for the need to isolate patients with exanthema ahead of establishing a diagnosis. Training programmes should include pictures of rashes to identify measles patients, an emphasis on the necessity for proper history taking, as well as considerations of the possible pitfalls of patients with atypical presentations. Finally, our findings suggest thorough testing of all patients with symptoms suggestive for measles. The costs for testing were relatively small compared with costs related to not recognised cases and/or diagnostic delay.
